# Intracardiac Ultrasound and Mapping Integration-Guided Endomyocardial Biopsy for the Diagnosis of Myocardial Melanoma Metastasis

**DOI:** 10.1016/j.jaccao.2024.01.008

**Published:** 2024-03-19

**Authors:** Jerry Fan, Christopher Perez, Robert J. Widmer, Vinh Nguyen, Gang Zhou, Laith Wahab, Javier E. Banchs

**Affiliations:** a Division of Cardiology, Baylor Scott and White Health-Temple, Temple, Texas, USA; b Department of Pathology, Baylor Scott and White Health-Temple, Temple, Texas, USA

**Keywords:** cardiac mapping, endomyocardial biopsy, intracardiac ultrasound, melanoma

**Figure:**
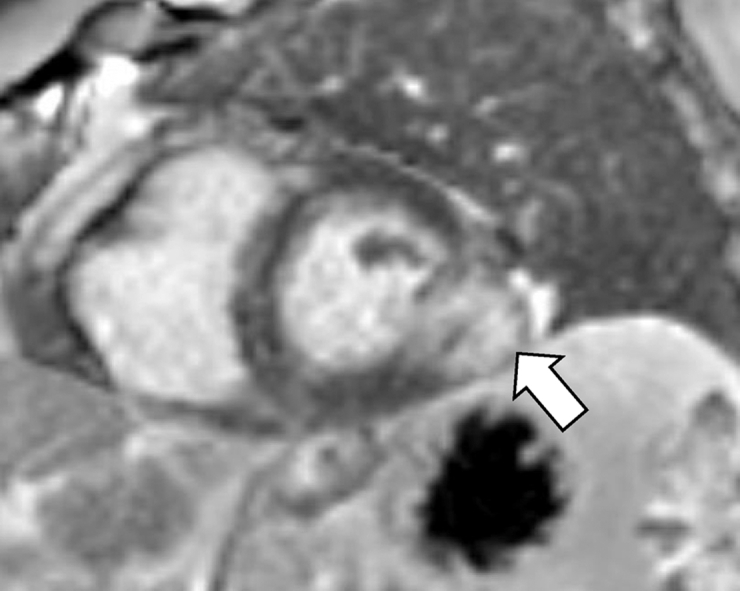

Metastatic melanoma is a highly aggressive malignancy with a poor prognosis. Cardiac metastases from melanoma are rare but can lead to significant morbidity and mortality. The diagnosis of cardiac metastases from melanoma can be challenging because of the lack of specific imaging modalities.^1^^,^^2^

Endomyocardial biopsy (EMB) can provide a definitive diagnosis of cardiac metastases, but it is an invasive procedure that carries a risk of complications. EMB has been used for many years to diagnose myocardial diseases.^3^, ^4^, ^5^, ^6^ However, the diagnostic yield of EMB can be limited because of the random sampling of the myocardium.^7^ The sensitivity of EMB varies depending on the underlying disease and the location of the biopsy.^3^, ^4^, ^5^ In some cases, multiple biopsies may be necessary to establish a diagnosis.^5^^,^^7^

Electroanatomic voltage mapping (EAVM) is a noninvasive imaging modality that can help guide EMB to increase its diagnostic yield and decrease the risk of complications.^5^, ^6^, ^7^ The use of EAVM in cardiac electrophysiology has been well established over the past decade.^6^^,^^7^ EAVM can provide accurate and detailed mapping of myocardial tissue by measuring the local electrical voltage.^5^, ^6^, ^7^ This allows for the identification of areas of scar or abnormal tissue that can guide the placement of diagnostic or therapeutic catheters.^5^, ^6^, ^7^ The use of EAVM in guiding EMB for the diagnosis of cardiac metastases from melanoma has only been reported in case reports and case series.^6^ In this clinical case challenge, we present our experience on the use of EAVM-guided EMB for the diagnosis of cardiac metastases from melanoma. Here an 86-year-old man with a history of metastatic melanoma with a recently discovered cardiac mass on a routine surveillance computed tomography scan was treated with a multidisciplinary team approach using EAVM-guided EMB to provide a timely and accurate diagnosis of his cardiac mass to plan further therapeutic options.

## Case Description

**Figure 1:**
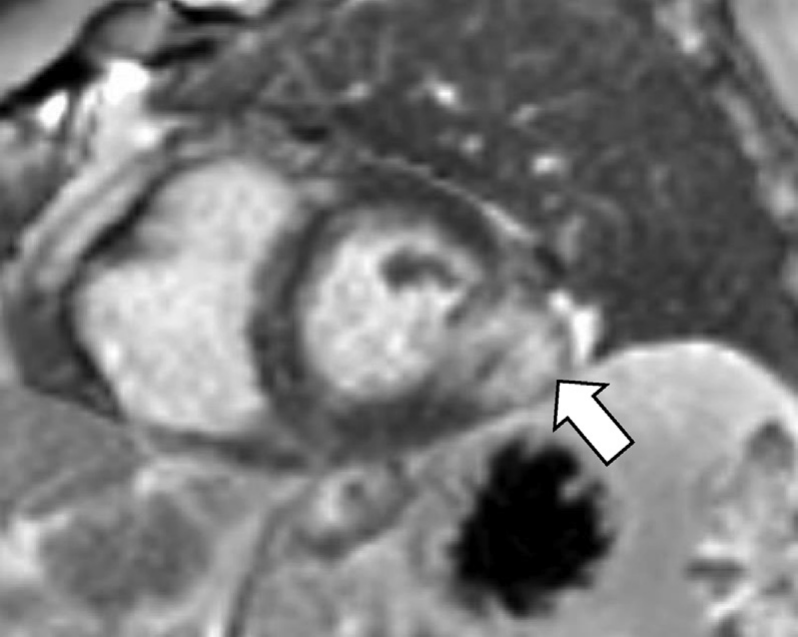
Cardiac Magnetic Resonance Imaging The figure illustrates the cardiac tumor and its characteristics. The arrow is pointing to the tumor that corresponds to an 18 F-fluorodeoxyglucose avid mass previously identified by positron emission tomography/computed tomography.

An 86-year-old man with a history of metastatic melanoma, previously in the right middle lobe of the lung treated with lobectomy and nivolumab for 6 cycles but was stopped because of significant side effects and subsequently monitored, was found to have an intracardiac mass on surveillance computed tomography. No cardiac-related symptoms or arrhythmias were present. Positron emission tomography/computed tomography showed the tumor was fluorodeoxyglucose avid. Cardiac magnetic resonance confirmed an exophytic mass measuring 3.3 × 2.2 cm at the midlateral segment extending through all 3 layers of the myocardium and abutting the pericardium. The mass demonstrated hyperintensity on steady-state free precession frequency and T1-weighted double inversion recovery sequences. T2-weighted imaging triple inversion recovery showed hyperintensity indicating the presence of edema. There was no significant gadolinium uptake on first-pass perfusion. There was late gadolinium enhancement (LGE) of the mass with a central core of hypoenhancement of an eccentric T2 hyperintense and T1 isointense left lateral ventricular tumor of 2.2 × 3.3 cm (Figure 1). To confirm the suspected diagnosis of myocardial metastatic melanoma and establish an appropriate treatment plan, a multidisciplinary cardiology team decided to perform a left ventricular endomyocardial biopsy guided by real-time imaging.

After consent from the patient and his family and consultation with oncology, the patient was taken to the electrophysiology laboratory for the biopsy. Electrodes for the Carto 3 (Biosense Webster) electroanatomic 3-dimensional mapping system were applied to the patient’s back, and the Sentinel Cerebral Protection System (Boston Scientific) was deployed in the right innominate and left carotid arteries after access was obtained to the right radial artery. Intravenous heparin was administered to anticoagulate the patient.

**Figure 2:**
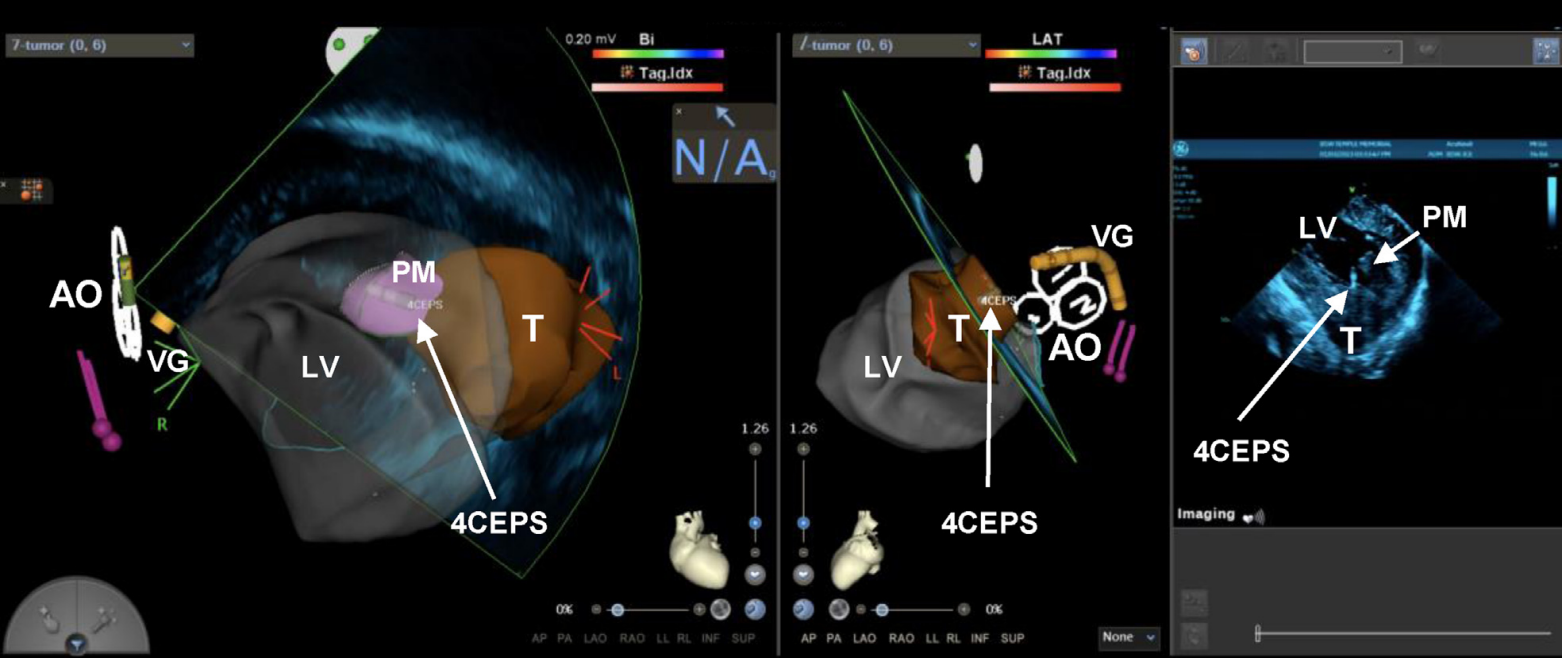
Carto Mapping System Screen During Endomyocardial Biopsy The figure is taken from the Carto electroanatomic voltage mapping system illustrating 3-dimensional anatomical geometry created using Cartosound (integration of real-time intracardiac ultrasound images superimposed to an anatomical matrix). Geometry of the left ventricle (LV) in transparent gray, the cardiac tumor (T) in brown, and the anterolateral papillary muscle (PM) in pink is shown in the (left) anterior-posterior and (right) left lateral (right) projections. The aortic cusps (AO) are traced with white outline; the Vizigo deflectable sheath (VG) is represented (mustard color) with spatial representation of the biopsy forceps (4CEPS). An intracardiac echocardiography frame is depicted showing the LV, lateral PM, and 4CEPS directed over the cardiac T. Arrows point to the corresponding anatomical structures of the abbreviation.

The right femoral vein was accessed to advance an 8-F sensor-enabled intracardiac ultrasound catheter (Soundstar, Biosense Webster) into the right cardiac chambers where the absence of intracardiac thrombus was confirmed. The Cartosound software (Biosense Webster) was used to register and reconstruct the left ventricular anatomy, and the tumor and anterolateral papillary muscle were tagged (Figure 2).

Transseptal access was obtained with a Baylis VersaCross sheath and electrified wire under intracardiac ultrasound guidance. The sheath was replaced with a Vizigo deflectable sheath (Biosense Webster), which was advanced into the left ventricle. A matrix was created with a PentaRay NAV multielectrode catheter (Biosense Webster) to visualize other electrified tools in the spatial registration of the left ventricular cardiac anatomy. Intracardiac bipolar voltage recorded from the endocardium overlying the tumor was normal (>1.5 mV).

A biopsy forceps was connected to a bipolar cable and advanced through the Vizigo deflectable sheath. The forceps was directed to the tumor with the aid of the deflectable sheath and intracardiac ultrasound visualization, and multiple samples were obtained and sent to pathology for evaluation (Figure 2). The patient tolerated the procedure well, and all catheters were removed. Heparin was reversed with intravenous protamine, and the patient returned to recovery. A follow-up echocardiogram showed no evidence of pericardial effusion, and the patient had no evidence of neurologic deficit. Aspirin 81 mg daily was prescribed for 1 month. Because the tumor did not invade into the left ventricle, long-term anticoagulation was not considered indicated.

Pathology confirmed cardiac melanoma metastasis, and the patient was counseled and scheduled for immunotherapy with an intravenous nivolumab and relatlimab combination. Follow-up imaging demonstrated a reduction in size from 3.3 × 2.2 cm to 2.6 × 1.4 cm after 6 months of treatment.

## Case Discussion

Obtaining a tissue diagnosis of myocardial disease that is infiltrative, inflammatory, or malignant often requires a cardiac biopsy; current guidelines suggest the use of EMB is reasonable if the diagnosis cannot be made without biopsy, will alter the treatment course, has a high success rate, and will be performed by an experienced operator.^3^, ^4^, ^5^^,^^7^^,^^8^ We suggest the use of EAVM-guided biopsy in cases of metastatic cancer in which tissue diagnosis and molecular studies would allow for more targeted therapy management.^3^, ^4^, ^5^^,^^7^^,^^8^ This can be done percutaneously through a transvenous sample or with a surgical approach.^3^, ^4^, ^5^^,^^9^ The transvenous EMB is a lower-risk procedure that is better tolerated, but its yield depends on the extent of the disease and the ability to direct the biopsy forceps into the affected area.^3^, ^4^, ^5^^,^^7^, ^8^, ^9^ Imaging modalities such as echocardiography and electroanatomic mapping systems have been used to guide the procedure, especially in cases of uncommon diseases such as sarcoidosis, eosinophilic myocarditis, or arrhythmogenic cardiomyopathy.^3^^,^^4^^,^^6^^,^^7^^,^^9^ Transthoracic echocardiography and positron emission tomography/computed tomography can identify morphologic and 18 F-fluorodeoxyglucose avidity, respectively.^3^^,^^4^^,^^9^ Cardiac magnetic resonance provides myocardial tissue characterization and in our case provided insights into the lesion characteristics (Figure 1). Cine steady-state free precession frequency assesses morphologic and dynamic motion with high spatial and temporal resolution. T1- and T2-weighted sequences discriminate the neoplastic lesion from a normal myocardium and are influenced by different tissue composition such as fat, edema, proteinaceous content, or hemorrhage. Unexpectedly, there was a lack of first-pass perfusion. We suspect this was caused by an abundance of tissue necrosis and edema and paucity of vascularity. There was intense LGE indicating the presence of inflammation and tissue necrosis/fibrosis. We suspect the well-circumscribed core of hypoenhancement on LGE imaging represented a focus of fluid.

In some cases, surgical or fluoroscopically guided EMB may have prohibitive risks.^3^^,^^4^^,^^8^^,^^9^ In such cases, the use of mapping systems and intracardiac ultrasound guidance can provide enhanced diagnostic yield and safety for the EMB.^5^, ^6^, ^7^, ^8^^,^^10^ For example, in a case in which a cardiac tumor affecting the lateral wall of the left ventricle required sampling, a transseptal access of the left ventricle was obtained using intracardiac ultrasound guidance.^3^^,^^4^^,^^9^^,^^10^ The tumor and anatomy were then identified and registered with intracardiac ultrasound in a 3-dimensional mapping system.^3^^,^^4^^,^^9^^,^^10^ A multielectrode catheter was used to register a map of the left ventricle, and a deflectable sheath with electrodes and a forceps connected as a quadripolar catheter to the mapping system allowed for alignment and visualization of the tumor, sheath, and forceps in the mapping system and ultrasound in real time during the specimen collection.^3^, ^4^, ^5^^,^^7^^,^^9^

The use of EAVM-guided EMB has been associated with an increase in diagnostic yield compared to traditional blind biopsy.^5^, ^6^, ^7^ EAVM-guided biopsy can provide detailed information on the location and extent of myocardial abnormalities, allowing for targeted biopsy sampling.^5^, ^6^, ^7^ This technique may be particularly useful in patients with suspected cardiac metastases from melanoma in whom blind biopsy may miss the affected areas.^6^ EAVM-guided biopsy may also reduce the need for multiple biopsies, which can be time-consuming and increase the risk of complications.^5^, ^6^, ^7^

However, the use of EAVM-guided biopsy requires specialized expertise and equipment, which may limit its widespread adoption.^5^, ^6^, ^7^ The procedure is also more complex and time-consuming than standard biopsy, and the diagnostic yield may still be limited in cases in which the underlying disease is diffuse.^5^, ^6^, ^7^ Nonetheless, EAVM-guided biopsy is a promising technique that can increase the diagnostic yield of biopsy and may be especially useful in cases in which traditional blind biopsy is not effective.^5^, ^6^, ^7^ Further studies are needed to evaluate the long-term clinical outcomes of EAVM-guided biopsy and its cost-effectiveness compared to traditional blind biopsy.

## Conclusions

EMB guided by intracardiac ultrasound integrated to a 3-dimensional electroanatomic mapping system is feasible and may result in higher yield and enhanced safety. In the case we present, the challenge represented by the location of a single lesion of a limited size could be effectively overcome with a percutaneous procedure, resulting in a definitive diagnosis.

## Funding Support and Author Disclosures

Dr Widmer is an advisor to Abbott, Medtronic, and Philips. All other authors have reported that they have no relationships relevant to the contents of this paper to disclose.
